# Protective Role of Endogenous Ovarian Hormones Against Learning and Memory Impairments and Brain Tissues Oxidative Damage Induced by Lipopolysaccharide

**DOI:** 10.5812/ircmj.13954

**Published:** 2014-03-05

**Authors:** Masoume Pourganji, Mahmoud Hosseini, Mohammad Soukhtanloo, Hoda Zabihi, Mosa Al-reza Hadjzadeh

**Affiliations:** 1 Neurocognitive Research Center, School of Medicine, Mashhad University of Medical Sciences, Mashhad, IR Iran; 2 Department of Biochemistry, School of Medicine, Mashhad University of Medical Sciences, Mashhad, IR Iran; 3 Department of Biology, Faculty of Biological Sciences, Kharazmi University of Tehran, Tehran, IR Iran; 4 Neurogenic Inflammation Research Center, School of Medicine, Mashhad University of Medical Sciences, Mashhad, IR Iran

**Keywords:** Learning, Memory, Ovariectomy, Lipopolysaccharide, Oxidative Stress

## Abstract

**Background:**

The contribution of neuroinflammation in Alzheimer’s disease (AD) has been widely reported. The effects of female gonadal hormones in both neuroinflammation and brain cognitive functions have also been well considered.

**Objectives:**

In the present study, the possible protective role for endogenous ovarian hormones against learning and memory impairment as well as brain tissues oxidative damage induced by lipopolysachride (LPS) was investigated in rats.

**Materials and Methods:**

The rats were divided into four groups: Sham-LPS, Ovariectomized (OVX)-LPS, Sham, and OVX. The animals of sham group were in proestrous phase in which the serum concentration of estradiol is high. The Sham-LPS and OVX-LPS groups were treated with LPS (250 µg/kg) before acquisition. The animals were examined using passive avoidance (PA) test. The brains were then removed and malondialdehyde (MDA) and total thiol groups concentrations were measured.

**Results:**

The time latency to enter the dark compartment by OVX-LPS group was shorter than that of OVX at both first and 24th hours after the shock (P < 0.05 - P < 0.001). In Sham-LPS and OVX-LPS groups, total thiol concentration in hippocampal and cortical tissues were significantly lower while MDA concentrations were higher than that of Sham and OVX groups (P < 0.05 - P < 0.001). ). The hippocampal MDA concentration in OVX-LPS group was higher than Sham- LPS group (P < 0.01).

**Conclusions:**

Brain tissue oxidative damage contributed in deleterious effects of LPS on learning and memory. Some protective effects for the endogenous ovarian hormones against damaging effects of LPS on learning and memory function, as well as brain tissues oxidative damage could be postulated; however, it needs more investigation.

## 1. Background

Alzheimer’s disease (AD) is a common type of dementia and about 13 million people suffer from it worldwide. This degenerative disorder is accompanied by a progressive impairment in memory, cognition, and emotional processes and finally, it leads to social or occupational disability (1, 2). Preservation of cognitive functions and individual capability, slowing the progression of the symptoms, and improving the quality of life are the main treatment strategies in AD (3). In addition to the well accepted role of estrogen in female reproductive system, some studies have indicated their effects in the central nervous system (CNS) in regions that are crucial for learning and memory performances (4, 5). The presence of intracellular and membrane receptors of estrogen in several different areas of the brain including hippocampus, provides another evidence for this idea (5). It is documented that estrogen improves prefrontal cognitive functions such as working memory and attention (6). In addition, the improving role of estrogen in long term potentiation (LTP) in the hippocampus has been well documented (7). It is of interest that studies on proestrus phase in rats, when the peak level of serum estrogen happens, showed LTP and spine density enhancement in hippocampus (7, 8). Many reports have indicated that estrogen affects the spine density in the hippocampus by N-methyl-D-aspartic acid (NMDA) neurotransmission and increasing the NMDA receptor binding (9, 10).

Inflammation is a response of body to the injury. The essential role of immune system in the regulation of tissue homeostasis and the response to infection and injury is explained via microglia that regulates the innate immune system in the CNS (11, 12). They are also the main source of cellular mediators of neuroinflammatory processes (11, 13). They persistently produce agents that affect surrounding astrocytes, neurons, and other cellular components of inflammation such as chemokines and cytokines (13). Significant evidence achieved over the past decade has supported the findings that suggest a clear link between neuroinflammation and AD pathology (13, 14). It is suggested that activation of microglia is followed by an increased in expression of some proinflammatory cytokines such as interleukin-1β (IL-1β), interleukin-6 (IL-6), and tumor necrosis factor α (TNFα) in cell surface (15, 16). It was also shown that certain drugs with anti-inflammatory properties can improve memory performance (17, 18). Lipopolysaccharide (LPS) is one of the main parts of the outer membrane of gram-negative bacteria (19). It is suggested that neutrophils and macrophages respond to LPS and release IL-1β and TNF-α (19). Activation of these cells by LPS has been linked to the pathogenesis of neuronal death, neurogenesis failure, and hippocampus-dependent memory and synaptic plasticity impairments; however, the mechanisms for these effects are not well understood (20). The brain tissues oxidative damage has been considered as an important contributor in memory impairment by LPS (21). It has been illustrated that estrogen has powerful anti-inflammatory effects. A high level of circulating estrogens has been shown to improve inflammatory-related diseases (22). In addition, it has been reported that estrogen can reduce production of some pro-inflammatory cytokines via activation of its receptors such as receptor α (23). Furthermore, antioxidant properties of estrogens have been well-documented using in vitro and in vivo models (24-27). The protective effects of estrogen against free radical generators and excitotoxicity have also been reported. The beneficial effects of estrogen on learning and memory have been frequently attributed to its protective properties against oxidative damage (27).

## 2. Objectives

Regarding the effects of ovarian hormones on learning and memory in one hand and on neuroinflammation, oxidative stress on the other hand and with consideration of the effects of LPS on learning and memory, the possible protective role for endogenous ovarian hormones against learning and memory impairment as well as brain tissues oxidative damage induced by LPS was investigated in rats.

## 3. Materials and Methods

### 3.1. Animals and Drugs

Sixty female Wistar rats with 12 weeks of age (240 ± 10 g) were selected. The animals were housed in four to five per standard cages, at room temperature (22 ± 2˚C) on a 12 h light/dark cycle. Food and water were available ad libitum properly. Animal handling and all related procedures were approved by the Mashhad Medical University Committee on Animal Research. The animals were divided into four groups: Sham (n = 20), Ovariectomized (OVX) (n = 10), Sham-lipopolysaccharide (Sham-LPS; n = 20), and ovariectomized-lipopolysaccharide (OVX-LPS; n = 10). In Sham and Sham-LPS groups, nine to ten animals in proestrous phase were selected and used for the behavioral studies. The animals in the Sham-LPS and OVX-LPS groups were treated by single injections of LPS (250 µg/kg; Ip) (21) 30 minutes before training in passive avoidance (PA) test. The animals of Sham and OVX groups received 1 mL/kg of saline instead of LPS. Ketamin was purchased from Alfasan Company (The Netherlands). LPS was purchased from sigma. (Sigma Chemical Co). Other chemicals, which were used to measure malondialdehyde (MDA) and total thiol concentrations, were purchased from Merck Company.

### 3.2. Surgery

Before surgery, the rats were permitted 15 days for acclimatization to the animal house. The animals were ovariectomized under ketamine anesthesia. Abdominal incision was made through the skin of the flank of the rats and ovaries and ovarian fats were removed. The same procedure was performed on the sham rats except the wound was closed without removing the ovaries (28).

### 3.3. Vaginal Cytology

Vaginal cytology investigation was performed in sham and sham-LPS groups to select the animals with proestrous stage for behavioral studies. The estrous cycle of the female rats is lasting four to five days and includes four phases, namely, proestrous, estrous, metaestrous, and diestrous phases. Proestrous phase is defined as the time when estrogen levels are very high and is characterized by the presence of primarily epithelial cells with large nuclei. In estrous phase, the typical cell pattern is the presence of primarily cornified epithelial cells. Metaestrous phase comprises cornified cells, primarily cornified cells, and sometime a few epithelial cells. Diestrous phase typical cell pattern consists of mainly leukocytes with a few number of nucleated epithelial and cornified cells in smears. To ensure that the female rats were cycling, vaginal cytology investigation was started one week before each experiment and was continued every day. The rats were held by a hand and lavaged with approximately 1 mL of saline. Slides were read using light microscopy and estrous categories were classified based on cytological characteristics (29). 

Passive avoidance test. Passive avoidance apparatus included light and dark compartments separated by a small removable door. The animals were familiarized with the apparatus for 5 min during two consecutive days. On a training trial, the rats were placed in light compartment facing away from the door that was located between the two compartments and the latencies to enter the dark compartment were recorded. When the rats were entered completely into the dark compartment, an electric shock (1 mA with the duration of 2 s ) was delivered to the floor of the compartment. The animals were then transferred to their cages. At one and 24 hours later, the rats were located in the light room and the latencies to enter the dark room as well as the times spent by the animals in dark and light compartments were recorded and defined as retention trial (30).

### 3.4. Biochemical Assessment

Finally, the rats were sacrificed and the cortical and hippocampal tissues were separated, weighed, and submitted to determine of total thiol (SH) groups and MDA concentrations. DTNB (2, 2'-dinitro-5, 5'-dithiodibenzoic acid) reagent, which reacts with the SH group, was used to determine the total thiol groups. The produced yellow complex has a peak absorbance at 412 nm (31). In brief, 50 μL of tissue homogenates was added to 1 ml Tris-EDTA (ethylenediaminetetraacetic acid) buffer (pH = 8.6) and the absorbance was read at 412 nm against Tris-EDTA buffer alone (A1). Then, 20 μL of 10 mM solution of DTNB was mixed with the solution and it was stored in room temperature for 15 minutes and the absorbance was read again (A2). The absorbance of DTNB reagent was also read as blank (B). Total thiol concentration (mM) was calculated as follows (Equation 1) (32):

Equation 1. 

*Total thiol concentration (mM) = (A2 – A1 – B) × 1.07 / 0.05 × 13.6*

MDA levels are as an index of lipid peroxidation. MDA reacts with thiobarbituric acid (TBA) as a TBA reactive substance (TBARS) and produces a red complex. Briefly, 1 mL of brain homogenates was added to 2 mL of a complex solution containing TBA/trichloroacetic acid (TCA)/hydrochloric acid (HCL) and it was then boiled in a water bath for 40 minutes. After reaching to the room temperature, the solution was centrifuged at 1000 g for 10 minutes. The absorbance was read at 535 nm (33, 34). The MDA concentration was calculated as follows (Equation 2). 

Equation 2. 

*C (M) = Absorbance / (1.56 × 10^5^)*

### 3.5. Statistical Analysis

All data were expressed as means ± SEM. The data were evaluated by one-way ANOVA and post hoc test. Differences were considered statistically significant when P < 0.05.

## 4. Results

### 4.1. Behavioral Results

**Figure 1.**
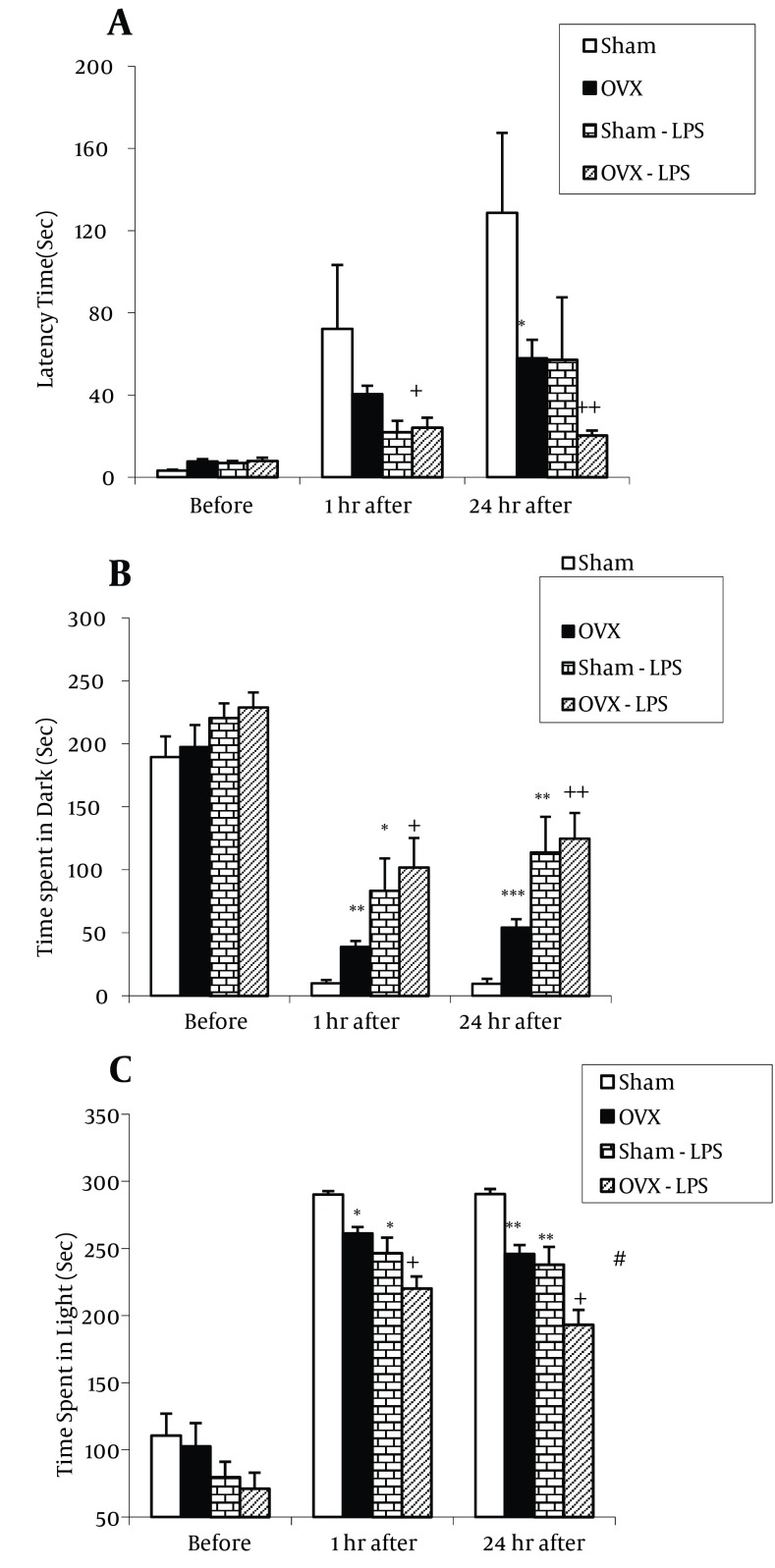
Comparison of Time Latency for Entering the Dark Compartment A. the total time spent in the dark compartment; B. the total time spent in the light compartment; C. at the first and the 24th hours after receiving shock in the experimental groups. Data are presented as Mean ± SEM (n = 9-10 in each group). The animals of Sham-LPS and OVX-LPS groups were treated by 250 µg/kg LPS before the training phase in passive avoidance test. The Sham and OVX groups were injected by saline instead of LPS. * P < 0.05, ** P < 0.01, *** P< 0.001 compared to Sham group, + P < 0.05, ++ P < 0.01 compared to the OVX group, # P < 0.05 compared to Sham-LPS group.

As shown in Figure 1 A, there was no significant difference between groups in time latency to enter the dark compartment before receiving shock. In OVX group, the time latency to enter the dark compartment at the first hour after receiving shock was not significant and at 24th hours after receiving shock was significantly lower than in Sham group (Figure 1 A, P < 0.05). The time latency to enter the dark compartment by the animals of OVX-LPS group was lower than that by OVX group at both the first and the 24th hours after receiving shock (P < 0.05, P < 0.01); however, there was no significant difference between Sham-LPS and Sham group. In addition, there was no significant difference between Sham-LPS and OVX-LPS group when the time latency was compared at the first and the 24th hours after receiving shock (Figure 1 A). When the total time spent in dark compartment were compared between four groups, there was no significant difference amongst them before receiving shock (Figure 1 B). The total time spent in dark compartment by the animals of OVX group was significantly longer than sham group at the first and the 24th hours after receiving shock (P < 0.01 and P < 0.001) (Figure 1 B). The animals of Sham-LPS group spent longer times in dark compartment in comparison with Sham group at the first and the 24th hours after receiving shock (P < 0.05 and P < 0.01) (Figure 1 B). The time spent by the animals of OVX-LPS group was significantly longer than that of OVX group at both the first and the 24th hours after receiving shock (P < 0.05 and P < 0.01 respectively) (Figure 1 B). Moreover, there was no significant difference between Sham-LPS and OVX-LPS group when the total time spent in dark compartment was compared at the first and the 24th hours after receiving shock (Figure 1 B). The Figure 1 C shows that there was no significant difference in the total time spent in light compartment before receiving shock. The total time spent in light compartment by the animals of OVX group was shorter than sham ones at both the first and the 24th hours after shock (P < 0.05 and P < 0.01) (Figure 1 C). The total time spent in light compartment in both sham and OVX rats treated by LPS was lower that none treated ones (P < 0.05 to P < 0.01) (Figure 1 C). The total time spent in light compartment in OVX-LPS group was lower than Sham-LPS group at the 24th hour after receiving shock (P < 0.05) (Figure 1 C).

### 4.2. Biochemical Results

**Figure 2.**
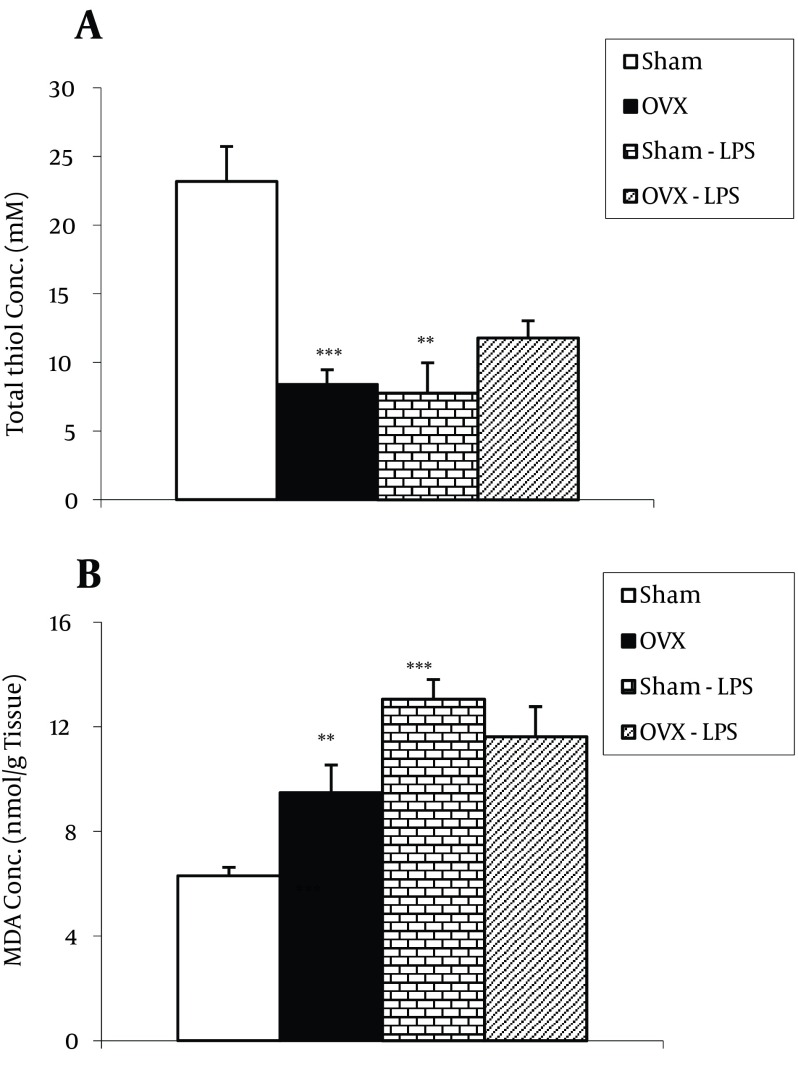
The Total Thiol Concentrations A. MDA concentrations; B. in cortical tissues of four groups. Data are shown as Mean ± SEM of 9-10 animals per group. ** P < 0.01, *** P < 0.001 compared to Sham group.

**Figure 3.**
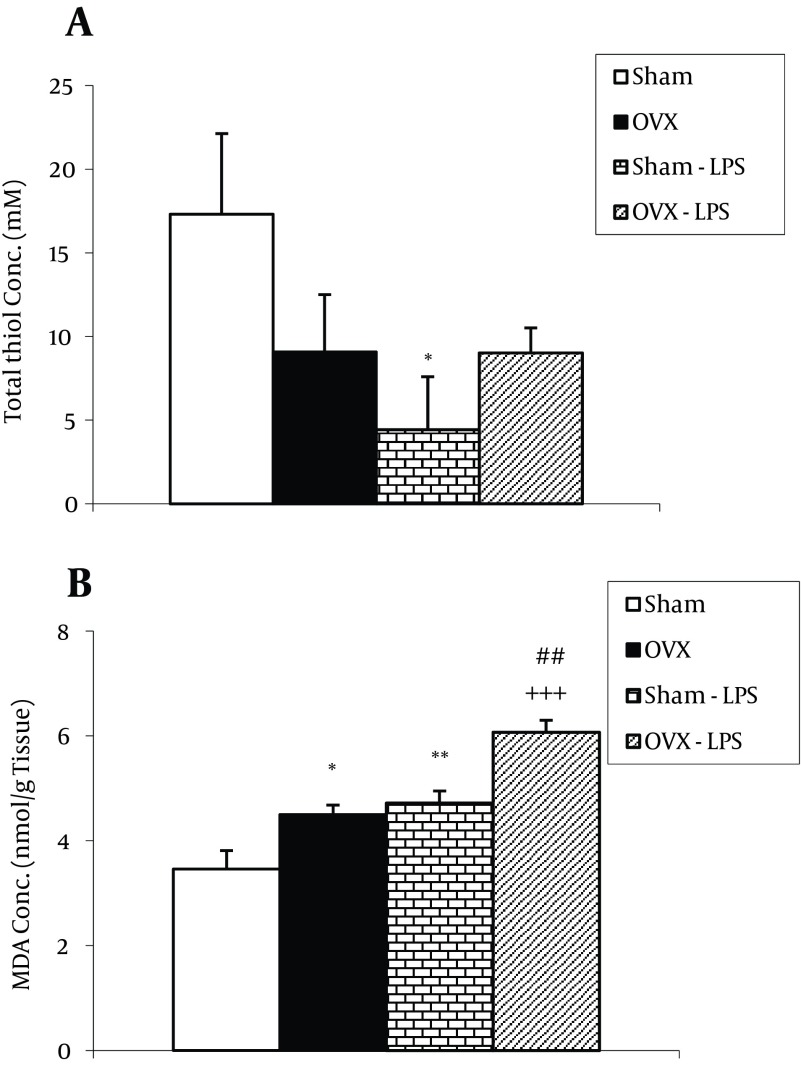
The Total Thiol Concentrations A. The MDA concentrations; B. in hippocampal tissues of four groups. Data are shown as Mean ± SEM of 9-10 animals per group. * P < 0.05, ** P < 0.01 compared to Sham group, +++ P < 0.001 compared with the OVX group, ## P < 0.01 compared to Sham-LPS group.

The total thiol concentration in cortical tissues of ovariectomized rats was significantly lower than sham animals (P < 0.001). In Sham-LPS group, the total thiol concentration was significantly lower than Sham group (P < 0.01). There was no significant difference between OVX-LPS and OVX groups in thiol concentration. Moreover, here was no significant difference between OVX-LPS and Sham-LPS groups (Figure 2 A). MDA concentration in cortical tissues of ovariectomized animals was higher than sham operated ones (P < 0.01). Injection of the sham-operated animals by LPS increased the MDA concentration in cortical tissues in contrast to sham group (P < 0.00) (Figure 2 B); however, there was no significant difference between OVX-LPS and OVX groups. There was also no significant difference between OVX-LPS and Sham-LPS groups (Figure 2 B). As Figure 3 A shows, the total thiol concentration in hippocampal tissues of ovariectomized rats was lower than that sham animals; however, it was not statistically significant. Treatment of the sham-operated rats by LPS significantly attenuated the total thiol concentration (P < 0.05); however, it was not effective in ovariectomized rats. In addition, there was no significant difference between OVX-LPS and Sham-LPS groups. The results showed that hippocampal MDA concentration in OVX group was significantly higher than that Sham group (P < 0.05) (Figure 3 B). MDA concentration in hippocampal tissues of both Sham-LPS and OVX-LPS groups was significantly higher than Sham and OVX groups (P < 0.01 and P < 0.001, respectively) (Figure 3 B). The hippocampal MDA concentration in OVX-LPS group was higher than Sham-LPS group (P < 0.01) (Figure 3 B). 

## 5. Discussion

The results of the present study showed that deprivation of ovarian hormones impaired learning and memory in rats. The results of passive avoidance test indicated that the time latency to enter the dark compartment by ovariectomized rats was shorter than sham operated rats. The total time spent in dark compartment by the animals of OVX group was also longer than by Sham group while the animals of OVX rats spent shorter time in light compartment in comparison to the sham-operated ones. The results of the present study confirmed the results of animals and human studies that reported learning and memory impairment by ovariectomy (28, 35-41). Co-localization of estrogen receptors as well as NMDA receptors in the hippocampus and neocortex might be another explanation for the role of estrogen in learning and memory (5, 42). Additionally, the interaction of estrogen with other neurotransmitters such as cholinergic and glutamate system has been considered. Another possible mechanism by which the ovariectomy influences learning and memory might be the decreases in NMDA receptor binding and/or calcium signaling pathways in hippocampal CA1 dendrites (43). In contrast to our results, no effect of estrogen status on learning and memory or even negative effects were reported (44, 45). in addition to multiple pathways (46), estrogen seems to exert its memory-related effect via antioxidant properties which might also have a role in its neuroprotective effects (47-49). Removal or blocking of the phenolic hydroxyl group on the A-ring of the steroid eliminates the antioxidant as well the neuroprotective properties; therefore, the antioxidative effects of estradiol might be in part due to the phenolic hydroxyl group (50-54). The results of the present experiment demonstrated that after ovariectomy, thiol contents in cortical and hippocampal tissues were decreased and MDA concentration increased. Ovariectomy model has been widely used as an animal model to mimic post-menopausal pathophysiological changes in learning and memory (55, 56). Therefore, it is suggested that learning and memory impairments that were seen in ovariectomized rats might be in part due to the oxidative damage to the brain tissues. The cognitive impairments due to human menopausal conditions might be related to the oxidative damage to the brain. The results of previous studies have also confirmed that ovariectomy of animals or postmenopausal conditions in women increased lipid peroxidation in brain, erythrocytes, and plasma (57, 58).

The impressing effects of neuroinflammation on neurodegenerative disorders such as AD have been well documented (14, 59, 60). Neuroinflammation induced by LPS has been shown to cause neuronal death, neurogenesis failure, and hippocampus-dependent memory and synaptic plasticity impairments (19, 20, 61, 62). The results of the present study showed that both ovariectomized and sham-operated rats injected by LPS had memory impairments in passive avoidance test. The results of present study confirmed LPS-induced learning and memory impairments which had been repeatedly reported (63-65).The results also confirmed the role of neuroinflammation in neurodegenerative disorders such as AD (14, 59, 60). Many different cell types such as neutrophils and macrophages are activated in neuroinflammation conditions induced by LPS that leads to release of the inflammatory mediators including IL-1β and TNF-α (61, 66). The brain tissues oxidative damage has been considered as an important contributor in memory impairment by LPS (21, 67). The results of present study confirmed this hypothesis; total thiol concentrations decreased and MDA concentrations increased in brain tissues of both sham and ovariectomized rats treated by LPS. As it was discussed previously, endogenous estradiol has considerable improving effects on learning, memory, and cognition. On the other hand, its anti-oxidant effects has been well documented. Regarding these facts, we hypothesized that ovarian hormones might have a protective role against impairments of learning and memory, as well as brain tissues oxidative damage induced by LPS. In the present study, LPS impaired learning and memory in both presence and absence of the ovarian hormones. The results of passive avoidance test showed that the animals OVX-LPS group spent a shorter times in light compartment after receiving shock in comparison to Sham-LPS group; however, there were no significant differences between these two groups when latency time and total time spent in dark compartment were compared. The sham-operated rats with proestrous phase were examined. It has been well documented that the plasma estradiol is in the highest level in comparison to the other phases such (8, 29). Therefore, it might be postulated that this high physiologic level of estradiol might be protective against learning and memory impairments induced by LPS; however, it needs to be more investigated. It has been previously reported that estradiol has a protective role in oxidative damage (68). Tang et al. found that oxidative damage significantly increased in rats eight weeks after ovariectomy (69). It is illustrated that the cytoprotection of estradiol is mediated through the reduction of reactive oxygen species production and induction of cellular antioxidant genes (70). In the present study, treatment by LPS increased the brain tissues MDA and decreased thiol concentrations in both the absence and presence of ovarian hormones. Regarding the higher level of MDA concentrations in brain tissues of OVX-LPS group in comparison to Sham-LPS, it seems that high level of estradiol has a protective role against brain tissues oxidative damage induced by LPS; however, it needs to be more investigated. Finally, it is concluded that brain tissue oxidative damage has a role in deleterious effects of LPS on learning and memory. Some protective effects for the endogenous ovarian hormones against damaging effects of LPS on learning, memory, and brain tissues oxidative damage could be postulated however, it needs to be more investigated.
